# The lifestyle of Tuyuhun royal descendants: Identification and chemical analysis of buried plants in the Chashancun cemetery, northwest China

**DOI:** 10.3389/fpls.2022.972891

**Published:** 2022-08-22

**Authors:** Yongxiu Lu, Bingbing Liu, Ruiliang Liu, Hongen Jiang, Yishi Yang, Qinhan Ye, Ruo Li, Wenyu Wei, Guoke Chen, Guanghui Dong

**Affiliations:** ^1^MOE Key Laboratory of Western China’s Environmental System, College of Earth and Environmental Sciences, Lanzhou University, Lanzhou, China; ^2^Gansu Provincial Institute of Cultural Relics and Archaeology, Lanzhou, China; ^3^The Department of Asia, British Museum, London, United Kingdom; ^4^Department of Archaeology and Anthropology, University of Chinese Academy of Sciences, Beijing, China

**Keywords:** archaeobotanical analysis, oxygen isotope, ancient Tuyuhun Kingdom, Hexi Corridor, Tang Dynasty, geopolitical situation

## Abstract

The Tuyuhun Kingdom (AD 313–663) was one of the most famous regimes in northwest China during the early medieval period. However, the lifestyle and spiritual pursuit of their descendants who became allied with the Tang Dynasty remain enigmatic. The excavation of the Chashancun cemetery, a Tuyuhun royal descendant (AD 691) cemetery in the Qilian Mountains in northwest China, reveals a large amount of uncharred plant remains. These remains provided a rare opportunity to explore the geographical origin of the buried crops and their social implications. In total, 253,647 crops and 12,071 weeds were identified. Foxtail millet and broomcorn millet represent 61.99 and 30.83% of the total plant remains, with the rest being barley, buckwheat, beans, and hemp. The oxygen isotope and trace elements of the crop and weed remains suggest that broomcorn millet, foxtail millet, barley, buckwheat, and hemp were sourced from different regions. The assemblage of plant remains in the Chashancun cemetery suggests that millet cultivation played an important role in the livelihoods of Tuyuhun descendants, and the location of the elite Tuyuhun cemetery and multisources of different buried crops may reflect their memory of ancestors and homelands. This case study provides a unique perspective to understand the interactions among human subsistence strategy, geopolitical patterns, and local natural environments in northwest China during the late 7th century.

## Introduction

**FIGURE 1:**
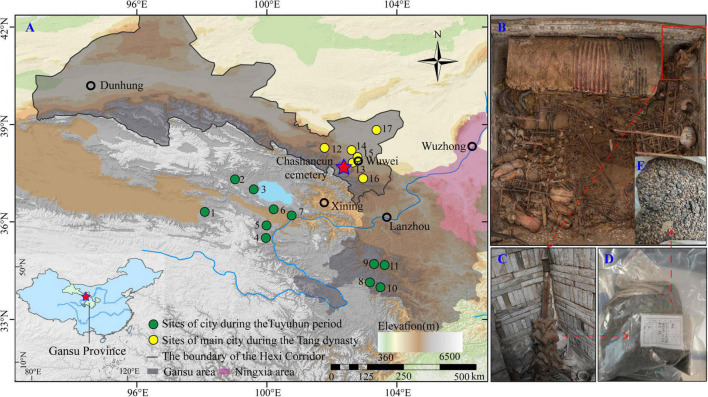
Location of the Chashancun cemetery **(A)** and the plant remains in the cemetery **(B–E)**. 1-Xiangride, 2-Jiamugeertan, 3-Tiebuqia, 4-Xiatang, 5-Zhidongjiala, 6-Qinghaihu, 7-Qugou, 8-Bugang, 9-Niutou, 10-Kaba, 11-Minghe, 12-Tianbao, 13-Guzang, 14-Jialin, 15-Shenniao, 16-Changsong, and 17-Wuwei.

One of the fundamental factors that underpinned the prosperity of the Tang dynasty was the close connection between the Central Plains of China and the Hexi Corridor. This connection supplied a range of important resources, technology, and manpower to both sides (Li, 1990b; Yang et al., 2020). While the importance of the Hexi Corridor was widely acknowledged in contemporary historical documents, less evidence can be found regarding the basic subsistence in the Hexi Corridor. The Tuyuhun Kingdom (AD 313–663) was one of the most powerful local regimes at the southern foot of the Qilian Mountains. The kingdom was established by the *Murong Xianbei* pastoralists at the end of the 3rd century, moving from the Yinshan mountains to the broader area of the Qaidam Basin, the Qinghai Lake Basin, and the upper Yellow River Valley (Escher, 2019; Cao, 2021). This rule lasted for 350 years, with 16 kings, until it was destroyed by the Tubo people in the third year of the *Longshuo* Tang Dynasty (AD 663) (Li et al., 2015; Pan, 2022). The rest of the Tuyuhun groups chose to ally with the Tang Empire (AD 618–907) after the fall of their own kingdom (Pu, 2017; Zhang, 2017; Niu, 2021). Most of the royal descendants of the Tuyuhun Kingdom migrated to Wuwei, the Hexi Corridor, and Wuzhong, Ningxia autonomous region (Figure 1A), but all of them chose to be buried in the Wuwei area after they died (Huang, 2017; Fan, 2018; Sha and Chen, 2021).

Understanding the subsistence strategy of these Tuyuhun royal descendants is of crucial archaeological and historical interest (Finkelstein and Perevolotsky, 1990; Chen et al., 2012; Farahani, 2018). According to historical documents such as *Sui Shu*, *Zi Zhi Tong Jian*, and *Tang Hui Yao*, the subsistence strategy of the Hexi Corridor was influenced by the expansion of the Tang culture. For example, the consumption of various crops, including foxtail millet, broomcorn millet, wheat, barley, buckwheat, hemp, pea, and black soya bean, was mentioned in these texts (Hao, 2011; Zhang, 2021). It is worth noting that the purpose of these documents is not exactly known to us; therefore, it is difficult to evaluate the accuracy of these records. Moreover, it is still difficult to extract any quantitative information from these fragmented texts and understand how different types of food were combined to support the local society.

The excavation of the Chashancun cemetery provides excellent archaeological records to resolve these important issues. A significant number of plant remains have been collected from four broken silk bags, which offered a rare opportunity to reveal the plant subsistence of the Hexi Corridor during the early Tang dynasty. Based on the identification of the plant remains, the analysis of oxygen isotopes and trace elements, and a survey through the historical documents, we aimed to reveal more detailed information on the subsistence pattern of the Tuyuhun descendants, examined single-source or multisource models of the different crop remains, and explored their broader social implication for the Tuyuhun royal descendants during the late 7th century.

## Study site

The Chashancun cemetery (102°22′54.3′′E, 37°40′51.7′′N) is located at the present-day Chashan village in the Qilian town (approximately 15 km from the city of Wuwei in the Gansu Province), Tianzhu Tibetan Autonomous County, which is 2,672 m above sea level. The average annual temperature and precipitation in this region are ∼1.6°C and 200–400 mm, respectively. As shown in Figure 1A, many settlements or cities during the Tuyuhun and the Tang periods were located at the southern and northern feet of the Qilian Mountains, respectively (Li, 1990a,^2014^). In contrast, the Chashancun cemetery was built on the top of the Qilian Mountain, most likely for the convenience of building and the spiritual pursuit of the ancient Tuyuhun Kingdom (Sha and Chen, 2021).

Numerous funerary objects including pottery, metalware, lacquerware, stone tools, silk fabrics, leather objects, sacrificial animals (i.e., horse and sheep), and food crops were discovered during the rescue excavation conducted by the Gansu Province Institute of Cultural Relics and Archaeological Research in 2019 (Gansu Provincial Institute of Cultural Relics and Archaeology et al., 2021). Based on the records of the epitaph, the Chashancun cemetery was owned by *Mu Rongzhi*, a Tuyuhun royal descendant, who died in the second year of *Tianshou* (AD 691).

## Materials and methods

Plant material is a significant component of funerary objects. Interestingly, they often appear to be carefully packaged or stored in small bags or pots to bury in the cemetery before deposition (Chen et al., 2012; Moustafa et al., 2018). The Chashancun cemetery is a single brick-room cemetery buried by dry arenosol on top of the Qilian mountains. The low permeability of the local soil and low precipitation create a favorable environment for the preservation of plant remains. Nine silk bags (*五谷囊 Wugunang*) containing desiccated and mixed plant remains were unearthed from the coffin chamber in the cemetery (Figures 1B,C). Unfortunately, four of them were damaged, and the plant remains were therefore transferred into new numbered sample bags during the excavation process (Figures 1D,E; Liu et al., 2022).

Given the large number of plant remains at Chashancun cemetery, three of the sample bags (the fifth, seventh, and ninth bags) were given to Lanzhou University for further research. These samples were separated and identified based on their morphological characteristics and compared with various illustrated identification keys (Guo, 1995; Guan, 2000; Liu et al., 2008). Meanwhile, a representative sample of each species was photographed under a stereomicroscope for high precision identification, and the percentage of certain species was calculated. Moreover, the poorly preserved samples were counted based on special features of the plant. For example, the count of hulled barley was based on the number of embryos of hulled barley. These tests were performed in the laboratory at the Lanzhou University and the University of Chinese Academy of Sciences.

As most of the plant remains identified from the Chashancun cemetery are well-preserved and uncharred, they were used for the analysis of oxygen isotope and trace element contents. A total number of 112 samples were processed in an automated hydrogen and oxygen analyzer linked to a Thermo Fisher Scientific (Bremen) GmbH Delta V Plus at the Key Laboratory of Western China’s Environmental Systems, Ministry of Education (MOE), Lanzhou University, China. In total, 30 element samples were measured in Inductively Coupled Plasma-Mass Spectrometry (ICP-MS) (Jena PQ MS) using the GB/T 14506.30-2010 method at the Createch Testing Technology Co., Ltd., Beijing, China. Before the samples of both experiments were measured, they were washed several times using distilled water in an ultrasonic cleaner for 30 min and then dried in an oven at 70°C for 12 h. They were subsequently crushed to powder using an agate mortar/pestle and weighed into tin containers. Each type of millet was divided into two groups (large vs. small) based on the morphological variations and then measured. More detailed criteria can be found in the study by Liu et al. (2022). The oxygen isotopic results were reported as δ^18^O relative to the international standard Vienna-Standard Mean Oceanic Water (VSMOW), and the isotopic analytical precision was 0.3‰. A total of 56 standards (IAEA-601) were entered into the oxygen isotopic sample list, and three standards (GSP-1, W-2a, AGV-2) were entered into the trace element sample list to monitor the data quality.

The principal component analysis (PCA) is one of the most commonly used multivariate statistical analysis methods that can reduce multiple dimensions embedded with the original data sets, which enables the new variables (the principal component) to be a linear combination of the original variables and provides a new dataset that can represent the original one (Wold et al., 1987; Abdi and Williams, 2010; Bro and Smilde, 2014). Meanwhile, cluster analysis is also applied to isolate homogeneous substrates among the items originating from heterogeneous substances based on certain characteristics of the observation (Krolczyk, 2014). Both methods can reveal the correlation and difference among the trace elements in plant remains cultivated from different areas. In this study, the PCA and cluster analysis were performed using the SPSS 26.0 software with the following steps: (1) standardize the original data; (2) determine the correlation between elements; (3) select the first two principal components; and (4) apply the cluster analysis.

## Results

### The assemblage of identified plant remains from the Chashancun cemetery

**FIGURE 2:**
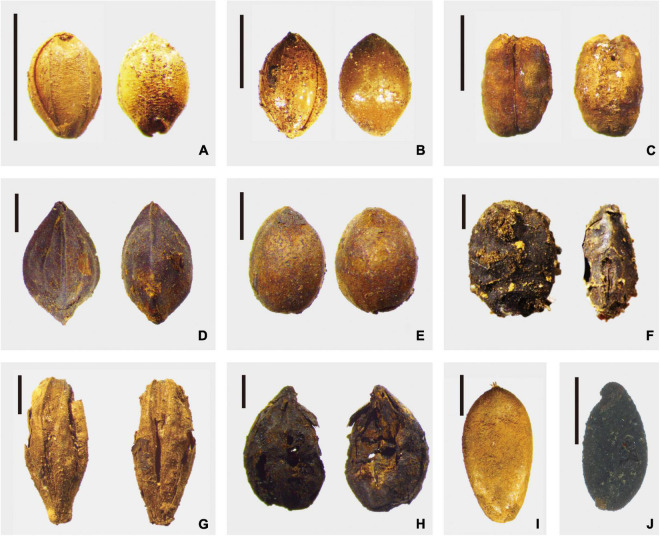
Crop remains unearthed from the Chashancun cemetery. **(A)** *Setaria italica*; **(B)** *Panicum miliaceum*; **(C)** *Triticum aestivum*; **(D)** *Fagopyrum esculentum*; **(E)** *Cannabis sativa sub. Sativa*; **(F)** *Leguminosae*; **(G)** *Hordeum vulgare*; **(H)** *Hordeum vulgare var. coeleste*; **(I)** *Cucumis melo*; and **(J)** *Linum usitatissimum* (Scale bar: 2 mm).

The Chashancun cemetery yielded a total of 265,718 uncharred plant remains, which can be divided into two primary groups, i.e., 253,647 crops and 12,071 weeds (Table 1). The plant remains that were identified from each sample bag and excavation notes suggest that the plants were mixed well and packed in nine silk bags before being buried in the cemetery (Supplementary Figure 1). Therefore, the plant remains in this study can be taken as representative of all the plant remains unearthed from the Chashancun cemetery. The six most important species were foxtail millet (*Setaria italica*), broomcorn millet (*Panicum miliaceum*), hulled barley (*Hordeum vulgare*), buckwheat (*Fagopyrum esculentum*), beans (Leguminosae), and hemp (*Cannabis sativa sub. Sativa*). Foxtail millet and broomcorn millet account for the highest proportion, being 61.99 and 30.82% of the total identified plant remains. Hulled barley, buckwheat, beans, and hemp represent relatively lower proportions of 1.82, 0.38, 0.22, and 0.16%. In comparison, other crops, such as naked barley (*Hordeum vulgare var. coeleste*), wheat (*Triticum aestivum*), flax (*Linum usitatissimum*), and muskmelon (*Cucumis melo*) played much smaller roles in the whole picture (all below 0.01%). The detailed morphological characteristics of each typical crop are shown in Figure 2. It is worth noting that all the plant remains were composed of chaff, probably due to the extended burial time.

**TABLE 1:** Summary data for the identified plant remains from the Chashancun cemetery.

Type of plant remains	Fifth bag	Seventh bag	Ninth bag	Sum	Proportion
**Crops**					
Foxtail millet	71,327	63,997	29,394	164,718	61.99%
Broomcorn millet	32,186	36,143	13,572	81,901	30.82%
Hulled barley	1,663	2,212	956	4,831	1.82%
Buckwheat	388	390	243	1,021	0.38%
Bean	235	218	138	591	0.22%
Hemp	190	152	84	426	0.16%
Naked barley	49	55	22	126	0.05%
Wheat	8	7	1	16	0.01%
Flax	5	7		12	–
Muskmelon	4	1		5	–
**Weeds**					
*Setaria glauca*	2,618	2,564	1,132	6,314	2.38%
*Chenopodium*	1,068	1,258	221	2,547	0.96%
*Echinochloa crusgalli*	1,002	1,102	356	2,460	0.93%
*Setaria viridis*	152	148	60	360	0.14%
*Vaccaria segetails*	116	95	43	254	0.10%
*Rubia cordifolia*	29	34	19	82	0.03%
*Humulus scandens*	15	15	2	32	0.01%
*Elymus*	6	2	4	12	–
*Compositae*	2	4		6	–
*Xanthium sibiricum*	2	1	1	4	–
**Aggregate**	111,065	108,405	46,248	265,718	100%

**FIGURE 3:**
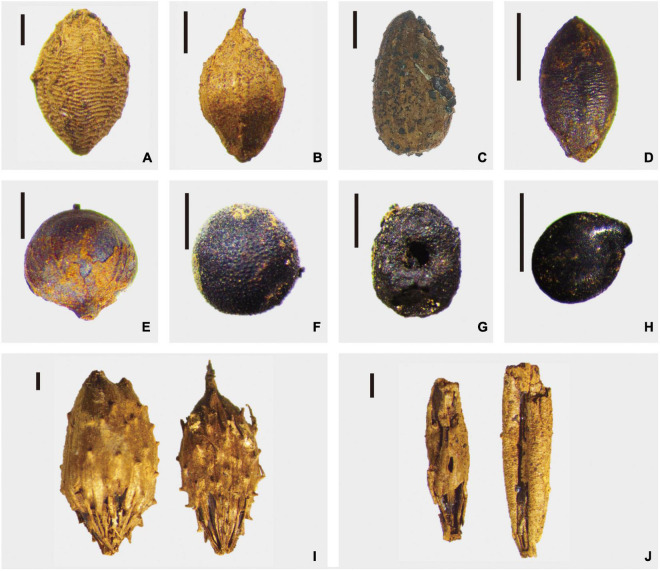
Weed remains unearthed from the Chashancun cemetery. **(A)** *Setaria glauca*; **(B)** *Echinochloa crusgalli*; **(C)** *Compositae*; **(D)** *Setaria viridis*; **(E)** *Humulus scandens*; **(F)** *Vaccaria segetalis*; **(G)** *Rubia cordifolia*; **(H)** *Chenopodium*; **(I)** *Xanthium sibiricum*; and **(J)** *Elymus* (Scale bar: 1 mm).

Ten species of weed plants were discovered, accounting for 4.54% of the total plant remains. As shown in Table 1, *Setaria glauca*, *Chenopodium*, and *Echinochloa crusgalli* occupied over 20% of the total weed plants, and several seeds of typical weed species, including *Setaria viridis*, *Vaccaria segetalis*, and *Rubia cordifolia*, show relatively higher proportions compared with *Humulus scandens*, *Elymus*, *Compositae*, and *Xanthium sibirium*. The morphological characteristics of typical weeds are shown in Figure 3.

### The oxygen isotope of different plant remains from the Chashancun cemetery

**FIGURE 4:**
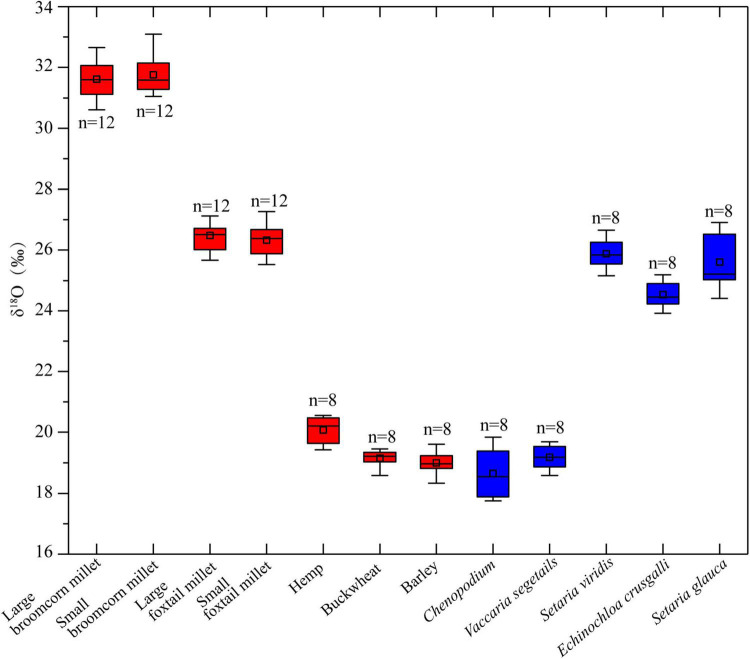
The box plot of oxygen isotopic values for plant remains from the Chashancun cemet.

The results of stable oxygen isotopic analysis of different plant remains are presented in Table 2 and Supplementary Table 1. The δ^18^O values of foxtail millet (*n* = 24) and broomcorn millet (*n* = 24) range from 25.5 to 27.8‰ (mean = 26.4 ± 0.6‰) and from 30.6 to 33.1‰ (mean = 31.7 ± 0.6‰). The δ^18^O values of hemp (*n* = 8) range from 19.4 to 20.6‰ (mean = 21.1 ± 0.4‰), while the buckwheat (*n* = 8) and hulled barley (*n* = 8) ranges from 18.6 to 19.5‰ (mean = 19.2 ± 0.3‰), and 18.3 to 19.6‰ (mean = 19.0 ± 0.4‰), respectively. The seed remains, such as *Chenopodium*, *V. segetalis*, *S. viridis*, *E. crusgalli*, and *S. glauca*, have different δ^18^O values (Table 2). As shown in Figure 4, the δ^18^O values of crop remains can be divided into three groups. Foxtail millet and broomcorn millet belong in two different groups; hemp, hulled barley, and buckwheat belong in the same group.

**TABLE 2:** Summary results of δ^18^O for the plant remains from the Chashancun cemetery.

Type of plant remains	Number of samples	Range	mean ± SD
Large broomcorn millet	12	30.6−32.7	31.6 ± 0.6
Small broomcorn millet	12	31.1−33.1	31.8 ± 0.6
Large foxtail millet	12	25.7−27.8	26.5 ± 0.6
Small foxtail millet	12	25.5−27.3	26.3 ± 0.5
Hemp	8	19.4−20.6	21.1 ± 0.4
Buckwheat	8	18.6−19.5	19.2 ± 0.3
Hulled barley	8	18.3−19.6	19.0 ± 0.4
*Chenopodium*	8	17.8−19.7	18.7 ± 0.8
*Vaccaria segetails*	8	18.6−19.7	19.2 ± 0.4
*Seteria viridis*	8	25.2−25.7	25.9 ± 0.5
*Echinochloa crusgalli*	8	24.0−25.2	24.5 ± 0.4
*Setaria glauca*	8	24.4−27.0	25.6 ± 0.9

### The trace element content of different plant remains from the Chashancun cemetery

**FIGURE 5:**
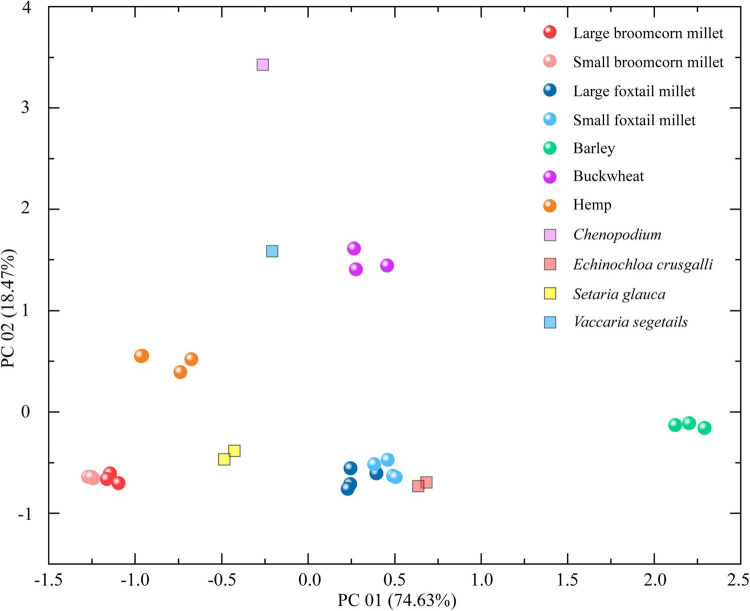
The scatter diagram of the first two principal components of trace element results for plant remains from the Chashancun cemetery.

**FIGURE 6:**
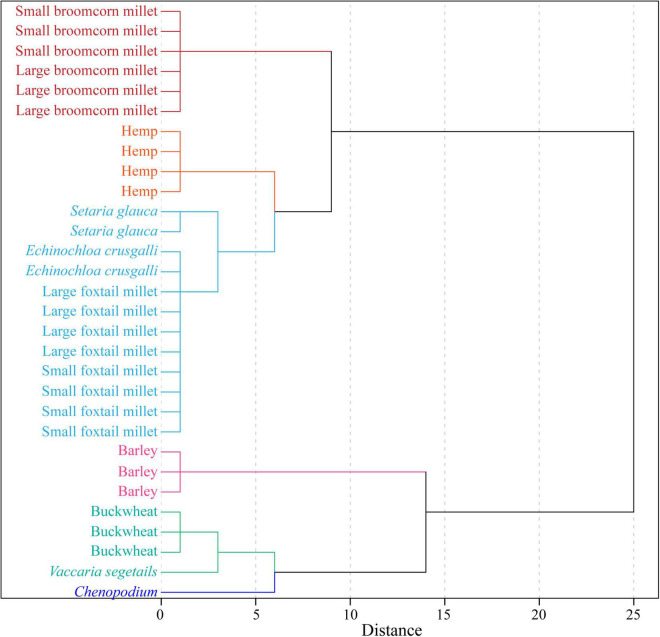
Element clustering analysis of plant remain samples from the Chashancun cemetery.

A total of 42 trace elements from 30 plant remain samples are presented in Supplementary Tables 2, 3. Principal component analysis was applied to the whole data set of plant remains. The first two principal components (PC 01 and PC 02) were selected, and they accounted for more than 93% of the total variation (Supplementary Tables 4, 5). This implies that these two components represent the most information from the original data and help reveal the potential outliers and trends in the data. As shown in Figure 5, the principal component score of different crops and weed remains displays a clear difference, especially between hulled barley and *Chenopodium*. The tree diagram illustrates the results produced by hierarchical clustering analysis. A smaller distance between clusters combined means more similarity. As the clustering distance increases, the data become more different from one another. Supplementary Table 6 and Figure 6 show that these 30 plant samples are clustered into two main groups when the clustering distance is set to 15 but change to six groups if the clustering distance is five. Broomcorn millet, hemp, hulled barley, and *Chenopodium* belong to four different groups, whereas buckwheat and *V. segetalis* belong to the same group. Foxtail millet, *S. viridis*, and *E. crusgalli* also belong to the same group.

## Discussion

### Plant subsistence of Tuyuhun royal descendants in the late 7th century

**FIGURE 7:**
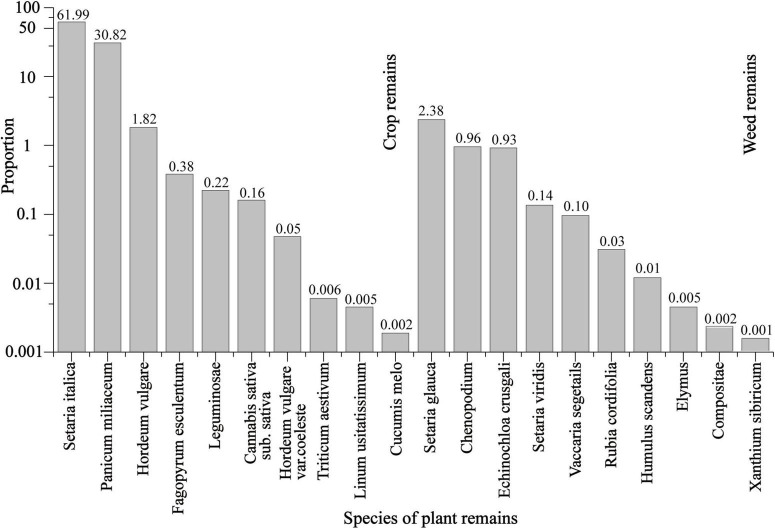
The proportion of different plant remains from the Chashancun cemetery.

Based on over 260,000 identified plant remains from the Chashancun cemetery (Table 1), foxtail millet and broomcorn millet were almost certainly the most important food consumed by the Tuyuhun royal descendants. Their total remain proportion reaches up to 97.23% (Table 1 and Figure 7). The proportion of hulled barley and buckwheat remains accounts for 1.90 and 0.40% of the overall crop remains, suggesting that they were important subsidiary crops for the Tuyuhun royal descendants during this period. Although hundreds of naked barley and bean grains were also identified from the Chashancun cemetery, their weights are much lower than other crops (Table 1 and Figure 7), probably suggesting that they might be used as auxiliary plant subsistence. The Tuyuhun royal descendants seem to use wheat, hemp, flax, and sweet melon as supplemental plant subsistence in the early period of the Tang Dynasty, as the proportion of these crop remains is all below 0.18%. Moreover, flax can be used for oil and fibers, and the muskmelon has a wide range of adaptations to the environment and can be used as an important source of fruit (Herbig and Maier, 2011; Reddy et al., 2013; Cao, 2021; Karg, 2022). Undoubtedly, the living resources of the indigenous inhabitants in the Hexi Corridor show extremely rich diversity during the early Tang dynasty.

Based on several historical records, millets were the dominant plant subsistence in the Dunhuang area of the western end of the Hexi Corridor during the early Tang Dynasty (Li, 1990b; Gao, 2017; Li et al., 2017). Other important contemporary crops cultivated in the same area included wheat, barley, buckwheat, beans, hemp, flax, and some vegetables and fruits (e.g., Shallot, Zingiber, radish, etc. and grape, peach, apricot, etc.) (Su, 2002; Hao, 2011). Moreover, few charred grains of wheat, barley, and foxtail millet were identified from the cultural layer dated to the Tang Dynasty in Datong City in the Hei River Basin (Shi, 2018). Various lines of evidence indicate that foxtail and broomcorn millet were dominant crops in the Hexi Corridor during the late 7th century, corresponding well with the agricultural system in central and northern China (Hua, 1990; Zhou and Garvie-Lok, 2015; Zhang, 2019; Zhang J. F. et al., 2021).

The oxygen stable isotopic composition has been widely applied to the study of the water source of both archaeological and modern crops (Kelly et al., 2002; Williams et al., 2005). According to the historical documents, the fact that the descendants of Tuyuhun lived in different areas of the Gansu and Ningxia regions (Zhang, 2017; Li et al., 2021) indicates that the buried crops were likely to derive from different regions as well. Given that all these regions are fairly desiccated, the majority of the changes in the plant δ^18^O could be attributed to soil water, which is ultimately influenced by local temperature and precipitation (Duan et al., 2008; Zhang et al., 2020). While no major effects on oxygen isotopes have been observed during plant uptake of soil water (Barbour, 2007), future studies remain critical to understand to what degree δ^18^O could be affected by the internal circulation of water (e.g., metabolism water stress caused by dry environment) in different species or regions. Meanwhile, the characteristics of the elemental composition of cultivated plants are associated with that of local soils and, therefore, can be used to differentiate the geographical origins of crops (Kelly et al., 2002; Sun et al., 2011; Zhou, 2011). As plant remains unearthed from the Chashancun cemetery are uncharred due to the special burial environment, different crop and weed remains are selected for analysis of oxygen isotopes and trace elements. The results are shown in Figures 4, 5.

The δ^18^O values of the broomcorn millet remains range between ∼31 and 32‰, and this is much higher than other crop and weed remains (Figure 4), suggesting that broomcorn millet might have been cultivated in a region different from other crops, which is further verified by the first two principal component contents (Figure 5). The δ^18^O values and the PCA of foxtail millet, *S. viridis* and *E. crusgalli* remains, both show clear overlap with each other (Figures 4, 5), suggesting that foxtail millet could have been cultivated in the same place with those weeds, probably symbiotic field with foxtail millet (Guan, 2000; Liu et al., 2008; Zhang et al., 2011). Although the principal components of *S. glauca* and foxtail millet are inconsistent, the cluster dendrogram shows that they might also be cultivated in the same area (Figure 6). In addition, we found that the difference in the principal components between large and small foxtail millet is relatively inconsistent compared with the broomcorn millet, and the δ^15^N values of the large foxtail millet fall in the range of the small foxtail millet (Liu et al., 2022). Therefore, it is more likely that foxtail millet may be cultivated in different places in one region.

The δ^18^O values of hulled barley, buckwheat, hemp, *V. segetalis*, and *Chenopodium* are all overlapped with one another (Figure 4), but the principal components of hulled barley, hemp, and *Chenopodium* are distinguished from the other plant remains (Figure 5). Similar to these results, cluster analysis shows that buckwheat and *V. segetalis* might have been cultivated together, and Hemp, Barley, and *Chenopodium* might have been cultivated in different places, respectively. Over 2,500 *Chenopodium* seeds were identified from three sample bags, which represent higher quantities compared with other crops, such as Buckwheat, Bean, and Hemp (Table 1 and Figure 7); the PCA and cluster analysis also show that there are distinct differences between *Chenopodium* and other plant remains (Figures 5, 6). However, *Chenopodium* unearthed from the Chashancun cemetery is more likely to have been weeds rather than crops by comparing it with domesticated *Chenopodium*, which often shows a large size with a thinner coat (Partap and Kapoor, 1985a,b, 1987; Xue et al., 2022). Therefore, it was most likely that *Chenopodium* growing in surrounding areas was mixed into crops during the crop processing and needs further verification. Considering that there are limited differences in water oxygen isotope on a regional scale during the same period (Bowen and Wilkinson, 2002; Lightfoot and O’Connell, 2016) and the elemental composition of soil varies significantly in different landscapes within a region (Robinson et al., 2009; Tudi et al., 2021), it can be inferred that broomcorn millet, foxtail millet, hulled barley, buckwheat, and hemp unearthed from the Chashancun cemetery might have been cultivated in different regions, whereas hulled barley, buckwheat, and hemp were probably cultivated in different places of one region. The exact location of cultivation for these crop remains is yet to be identified. However, it is not clear whether the isotopic fractionation of plant remains from the Chashancun cemetery existed during the diagenesis process (AD 691-2019) although the buried environment was fairly desiccated. Therefore, the conclusion based on oxygen isotope and trace element values needs to be examined in future studies.

### Influencing factors for plant remains buried in the Chashancun cemetery

The characteristics of plant remains identified from the Chashancun cemetery are evidently different from the agricultural structure of the ancient Tuyuhun people, who were primarily engaged in cultivating barley, millets, and beans before they lost their kingdom (Cao, 2021; Niu, 2021). Foxtail millet and broomcorn millet are overwhelmingly dominant crops in the Chashancun cemetery, which is consistent with the policy of the Tang Empire. The oasis in the Hexi Corridor was one of the most important centers for the cultivation of millet crops during the early Tang Dynasty (Hua, 1990; Li, 1990b; Li et al., 2017), and millets were used as the primary foodstuff in northern China during that time (Li, 2011; Zhang, 2019; Niu, 2021). While archaeobotanical studies suggest that barley and wheat were the most important crops in the Hexi Corridor during BC 1700-100 (Zhou et al., 2016; Yang et al., 2019; Dong et al., 2020), foxtail and broomcorn millet became the dominant crops in the Hexi Corridor since the Han Dynasty (BC 202-AD 220) (Gao, 2014; Shi, 2018; Li et al., 2020) when the central government in ancient China first controlled this area. These pieces of studies suggest that the policy of agricultural empires at that period might have profoundly influenced the cropping patterns in the Hexi Corridor, and also the predominance of millet crops in the buried plant remains unearthed from the Chashancun cemetery. The lifestyles of the elites of Tuyuhun had been evidently affected by the Tang Empire after their conversion to the Tang Empire (Escher, 2019; Niu, 2021; Pan, 2022), and *Mu Rongzhi* was one of the Tuyuhun royal descendants, who might have been significantly affected by the dietary habit of the Tang Dynasty. Consistent with the above processes, the primary crop was shifted from millet to wheat under the influence of the Tubo Empire in the Dunhuang area during AD 781–848 (Hao, 2011; Shi et al., 2022).

On a larger spatio-temporal scale, both the Tuyuhun and Turpan people, as the ancestors of Northwest Chinese, were finally conquered by the Han culture. But after a long time of interaction, the Tang civilization has deeply implanted these areas, for example, the shape and structure of tombs, the appearance of epitaphs, the use of characters, and the arrangement of burial goods (Li, 2014; Fan, 2018; Zhou, 2019). The grain remains found in this study include foxtail millet, broomcorn millet, hulled barley, naked barley, buckwheat, hemp, and a small amount of sweet melon and flax, which are similar to the arrangement of grains unearthed from the Astana cemetery in Turpan during the Jin and Tang dynasties (Chen et al., 2012). Based on the unearthed grains, the highly developed Tang civilization influenced the Tuyuhun tribe and the residents of Gaochang in Turpan. The assemblage of the buried crop remains in the Chashancun cemetery provides a vivid epitome that reflects the impact of geopolitical change on the livelihoods of Tuyuhun descendants during the 7th century.

The nostalgia for the ancient Tuyuhun Kingdom is another potential factor for the selection of buried plants in the Chashancun cemetery, which is proposed as a key reason to build the cemeteries for the Tuyuhun royal descendants on the top of the Qilian mountains (Zhou, 1994; Fan, 2018; Sha and Chen, 2021). In the same period of the Dunhuang area, wheat was the second most important crop (Hao, 2011; Gao, 2017), while barley was the most essential cultivated crop in the ancient Tuyuhun Kingdom (Cao, 2021; Niu, 2021). The memory of foodstuff and livelihoods is thought of as the inheritance of cultural identity for both individual and ethnic groups (Peng, 2013; Wallman, 2021). Therefore, the relatively high proportion of barley in relation to wheat may be the result of the inheritance of Tuyuhun descendants from the habits of their homeland. The selection of hemp as one of the buried crops in the Chashancun cemetery might be influenced by the custom of the ancient Tuyuhun. Hemp remains have been identified from the Yanghai Cemetery (BC 900-200) in Turpan of east Xinjiang Province, which might have been used for both medicine and shamanistic use (Zhao et al., 2019), which was also the faith of the ancient Tuyuhun kingdom (Sun, 2019; Cao, 2021). In addition, hemp was an essential crop during the Jin and Tang dynasties (Hao, 2011; Chen et al., 2012; Liu et al., 2017) and was one of the Five Grains buried in cemeteries during the Han Dynasty (Shen, 1998; Chen, 2015). Therefore, hemp in this study played an important role in the lives of local inhabitants during the early Tang Dynasty.

The local natural environment in the Hexi corridor and Ningxia areas, which were the primary settlement areas for Tuyuhun descendants, is probably another factor for the buried plants in the Chashancun cemetery. The average elevation of these areas is significantly lower than the territory of the ancient Tuyuhun Kingdom, such as the Qaidam and Qinghai Lake basins. It is advantageous to cultivate naked barley in the high-cold Tibetan Plateau, while hulled barley is more suitable to be cultivated in low-altitude plains for insect resistance (Capdevila et al., 1997; Yang et al., 2010). Thus, this natural environment of the Chashancun cemetery explains why the number of identified hulled barley grains is greater than the number of naked barley. Buckwheat was first domesticated and consumed in the eastern Hexi Corridor since Bronze Age (Wei, 2019; Zhang K. X. et al., 2021). The notable elevation difference and diverse landscapes on the northern side of the Qilian Mountain provide a favorable environmental foundation for the growth of the identified plant species from the Chashancun cemetery. Based on the oxygen isotope and trace element content of buried crops in the Chashancun cemetery, those crops were likely sourced from three different areas, probably from where the Tuyuhun descendants lived before death. This reflects the nostalgia for the second homeland of the Tuyuhun groups, which needs to be examined in future studies.

## Conclusion

Archaeobotanical analysis of the Chashancun cemetery in the Qilian Mountains provides new important physical materials to understand the plant subsistence and lifestyle of Tuyuhun descendants during the late 7th century. Millets were the predominant cultivated crops, and they were found with other important auxiliary plant subsistence, such as hulled barley, buckwheat, beans, and hemp. This reflects the integration of their dietary customs into the Tang Dynasty. However, broomcorn millet, foxtail millet, and other crops buried in the Chashancun cemetery were sourced from different regions due to the nostalgia for their living areas before death. The location of the Chashancun cemetery on the top of the Qilian Mountains, the hemp identified from the Chashancun cemetery, and the relatively high proportion of hulled barley indicate the deep memory of the ancestral lifestyles in the ancient Tuyuhun Kingdom, which also might be affected by the natural environment of the Hexi Corridor and Ningxia areas. More studies of various funerary objects from the Chashancun cemetery are needed to comprehensively understand the livelihoods and spiritual cares of Tuyuhun royal descendants.

## Data availability statement

The original contributions presented in the study are included in the article/Supplementary material, further inquiries can be directed to the corresponding authors.

## Author contributions

GD and GC designed this study. BL, YY, and WW conducted field surveys and sample collection. YL, HJ, and QY completed experiments and data correction.YL, BL, RL and WW analyzed data and designed the figures. GD, RLL, BL, and YL wrote the manuscript in consultation with all authors. All authors discussed the results, commented on the manuscript, and approved the submitted version.
