# Supplementary material for: The lifestyle of Tuyuhun royal descendants: Identification and chemical analysis of buried plants in the Chashancun cemetery, northwest China
Source: Front Plant Sci. 2022 Aug 22;13:972891. doi: 10.3389/fpls.2022.972891 (PMC9441944; doi:10.3389/fpls.2022.972891)
Supplement: Supplementary file 1 [file Data_Sheet_1.PDF]

Supplementary Material

1 Supplementary Figures and Tables

1.1 Supplementary Figures

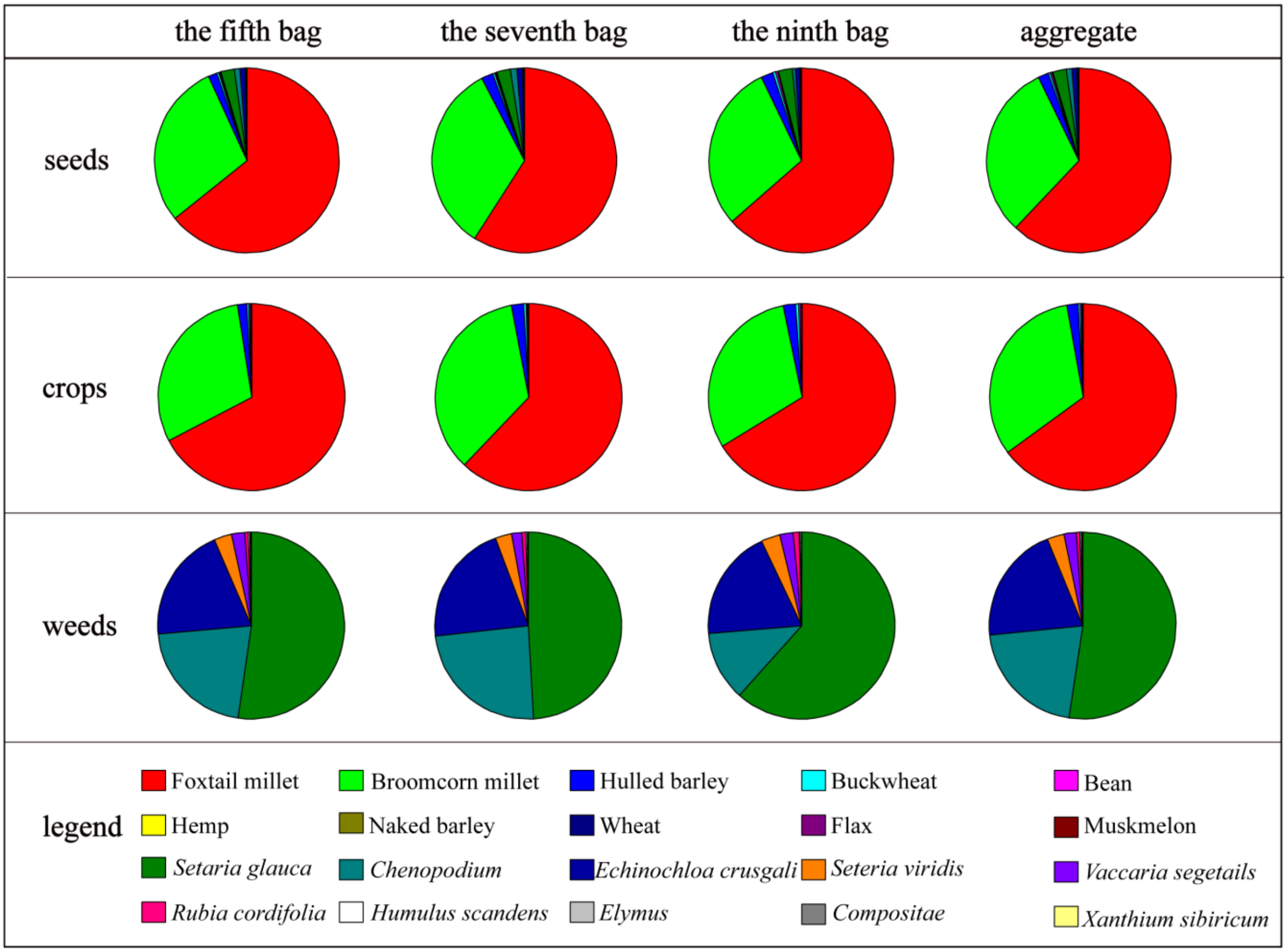

Supplementary Figure S1. The proportion of different plant remains of total remains from the Chashancun cemetery from three different bags.

1.2 Supplementary Tables

Table S1 The result of oxygen isotopic value for plant remains from the Chashancun cemetery.

Total umber	Number of per category	Samples	oxygen isotopic value

1	1	Large broomcorn millet	30.6
2	2	Large broomcorn millet	32.7
3	3	Large broomcorn millet	31.2
4	4	Large broomcorn millet	31.5
5	5	Large broomcorn millet	31.0
6	6	Large broomcorn millet	32.2
7	7	Large broomcorn millet	31.9
8	8	Large broomcorn millet	31.4
9	9	Large broomcorn millet	32.1
10	10	Large broomcorn millet	32.0
11	11	Large broomcorn millet	31.7
12	12	Large broomcorn millet	31.0
13	1	Small broomcorn millet	31.0
14	2	Small broomcorn millet	32.2
15	3	Small broomcorn millet	32.5
16	4	Small broomcorn millet	31.5
17	5	Small broomcorn millet	31.5
18	6	Small broomcorn millet	33.1
19	7	Small broomcorn millet	31.8
20	8	Small broomcorn millet	31.2
21	9	Small broomcorn millet	32.1
22	10	Small broomcorn millet	31.6
23	11	Small broomcorn millet	31.3
24	12	Small broomcorn millet	31.2
25	1	Large foxtail millet	27.8
26	2	Large foxtail millet	26.5
27	3	Large foxtail millet	26.5
28	4	Large foxtail millet	25.9
29	5	Large foxtail millet	26.7
30	6	Large foxtail millet	25.7
31	7	Large foxtail millet	26.2
32	8	Large foxtail millet	27.1
33	9	Large foxtail millet	26.7
34	10	Large foxtail millet	26.6
35	11	Large foxtail millet	25.7
36	12	Large foxtail millet	26.6
37	1	Small foxtail millet	26.4
38	2	Small foxtail millet	26.1
39	3	Small foxtail millet	26.5
40	4	Small foxtail millet	25.5
41	5	Small foxtail millet	26.8
42	6	Small foxtail millet	25.6
43	7	Small foxtail millet	26.6
44	8	Small foxtail millet	27.3
45	9	Small foxtail millet	26.3
46	10	Small foxtail millet	25.6
47	11	Small foxtail millet	26.3
48	12	Small foxtail millet	26.9
49	1	Hemp	19.7
50	2	Hemp	20.0
51	3	Hemp	20.4
52	4	Hemp	19.4
53	5	Hemp	20.5
54	6	Hemp	20.4
55	7	Hemp	19.6
56	8	Hemp	20.6
57	1	Buckwheat	19.4

58	2	Buckwheat	19.1
59	3	Buckwheat	19.3
60	4	Buckwheat	19.5
61	5	Buckwheat	18.9
62	6	Buckwheat	19.3
63	7	Buckwheat	18.6
64	8	Buckwheat	19.1
65	1	Barley	19.6
66	2	Barley	18.8
67	3	Barley	19.0
68	4	Barley	19.0
69	5	Barley	19.2
70	6	Barley	18.8
71	7	Barley	18.3
72	8	Barley	19.3
73	1	Chenopodium	19.5
74	2	Chenopodium	19.8
75	3	Chenopodium	19.3
76	4	Chenopodium	17.8
77	5	Chenopodium	17.9
78	6	Chenopodium	18.5
79	7	Chenopodium	17.9
80	8	Chenopodium	18.6
81	1	Vaccaria segetails	19.7
82	2	Vaccaria segetails	19.6
83	3	Vaccaria segetails	19.0
84	4	Vaccaria segetails	19.3
85	5	Vaccaria segetails	18.7
86	6	Vaccaria segetails	19.4
87	7	Vaccaria segetails	19.0
88	8	Vaccaria segetails	18.6
89	1	Seteria viridis	25.8
90	2	Seteria viridis	26.2
91	3	Seteria viridis	26.7
92	4	Seteria viridis	25.4
93	5	Seteria viridis	25.8
94	6	Seteria viridis	26.3
95	7	Seteria viridis	25.7
96	8	Seteria viridis	25.2
97	1	Echinochloa crusgalli	24.4
98	2	Echinochloa crusgalli	24.8
99	3	Echinochloa crusgalli	24.3
100	4	Echinochloa crusgalli	25.2
101	5	Echinochloa crusgalli	24.5
102	6	Echinochloa crusgalli	24.2
103	7	Echinochloa crusgalli	25.0
104	8	Echinochloa crusgalli	23.9
105	1	Setaria glauca	25.0
106	2	Setaria glauca	25.0
107	3	Setaria glauca	26.6
108	4	Setaria glauca	25.3
109	5	Setaria glauca	26.9
110	6	Setaria glauca	26.5
111	7	Setaria glauca	24.4
112	8	Setaria glauca	25.2

Table S2 The element results of plant remain from Chashancun cemetery.

Samples	Li	Be	Sc	Ti	V	Cr	Mn	Co	Ni	Cu	Zn	Ga	Rb	Sr	Y	Zr	Nb	Mo	Sn	Cs	Ba	La	Ce	Pr
**Large broomcorn millet**	0.14	0.040	0.16	30.0	2.95	1.46	33.7	0.78	4.49	32.6	45.5	0.27	0.77	19.7	0.17	1.09	0.10	2.50	0.38	0.079	7.82	0.43	0.84	0.095
**Large broomcorn millet**	0.13	0.040	0.15	28.2	2.71	1.75	37.0	0.73	4.51	24.3	46.1	0.27	0.78	18.5	0.14	1.98	0.16	2.36	0.22	0.079	7.35	0.34	0.66	0.075
**Large broomcorn millet**	0.12	0.039	0.14	25.9	2.70	1.34	34.7	0.73	4.23	24.2	45.8	0.27	0.73	18.4	0.44	1.08	0.097	2.53	0.21	0.075	7.06	0.38	0.76	0.083
**Small broomcorn millet**	0.11	0.040	0.13	22.4	2.33	1.21	25.1	0.64	3.92	23.0	42.0	0.23	0.66	27.4	0.16	1.51	0.098	3.56	0.22	0.065	6.57	0.34	0.64	0.073
**Small broomcorn millet**	0.094	0.037	0.13	22.3	2.21	1.21	25.4	0.65	3.99	23.2	42.5	0.23	0.64	27.8	0.14	1.24	0.092	3.63	0.23	0.066	6.53	0.32	0.59	0.068
**Small broomcorn millet**	0.11	0.040	0.13	22.3	2.47	1.43	25.3	0.65	4.05	23.2	42.3	0.23	0.68	27.8	0.17	1.35	0.088	3.60	0.22	0.066	6.67	0.31	0.58	0.068
**Large foxtail millet**	0.81	0.10	0.52	131	7.11	5.43	93.0	1.79	9.80	52.0	104	0.78	3.70	57.2	0.95	5.96	0.44	5.88	0.73	0.31	28.2	1.45	2.83	0.31
**Large foxtail millet**	0.71	0.099	0.47	109	6.75	3.86	99.1	1.82	9.76	50.1	102	0.76	3.24	54.4	0.87	4.20	0.38	5.74	0.71	0.29	26.2	1.49	2.99	0.32
**Large foxtail millet**	0.74	0.093	0.42	128	7.14	4.13	86.6	2.37	9.74	54.2	104	0.19	3.72	60.5	1.06	6.62	0.40	5.26	0.76	2.67	23.3	1.35	2.65	0.30
**Large foxtail millet**	0.70	0.091	0.40	126	6.92	3.60	85.9	2.34	9.45	54.1	103	0.20	3.72	60.7	1.09	6.51	0.41	5.18	0.78	2.69	23.9	1.33	2.60	0.31
**Small foxtail millet**	0.81	0.10	0.53	127	7.02	4.16	108	1.91	10.2	57.3	109	0.81	3.69	59.2	0.99	5.41	0.44	5.61	0.78	0.85	29.6	1.68	3.27	0.36
**Small foxtail millet**	0.80	0.11	0.49	126	7.13	3.81	107	1.89	10.1	57.3	109	0.82	3.67	59.4	1.04	5.19	0.44	5.68	0.77	0.89	29.3	1.77	3.48	0.38
**Small foxtail millet**	0.77	0.095	0.47	115	6.71	3.70	97.5	1.87	10.2	58.3	109	0.76	3.40	61.3	0.98	4.69	0.41	5.74	0.95	0.37	28.1	1.49	2.92	0.32
**Small foxtail millet**	0.80	0.099	0.43	116	6.85	4.71	115	2.08	11.4	58.6	116	0.82	3.64	63.4	1.07	4.90	0.44	5.65	0.73	0.36	29.1	1.58	3.06	0.33
**Barley**	1.25	0.16	0.79	211	11.2	7.38	201	4.52	20.6	106	219	1.41	6.07	113	2.20	10.7	0.83	9.28	1.13	0.48	52.7	2.91	5.56	0.62
**Barley**	1.21	0.15	0.73	206	11.4	6.66	185	4.44	20.3	107	222	1.35	5.72	108	1.95	16.7	0.81	9.57	1.10	0.46	49.2	2.69	5.43	0.61
**Barley**	1.21	0.16	0.73	212	10.8	6.59	188	4.45	20.4	109	220	1.36	5.87	111	1.91	12.8	0.86	9.80	1.10	0.46	51.5	2.64	5.30	0.59
**Buckwheat**	0.52	0.089	0.32	83.0	13.3	5.39	178	7.04	26.5	147	266	1.38	2.18	135	0.93	5.28	0.37	25.3	0.75	0.20	22.8	1.22	2.44	0.27
**Buckwheat**	0.49	0.085	0.30	79.9	13.3	5.35	156	6.39	25.8	152	266	1.25	2.00	131	0.83	3.43	0.36	25.3	0.69	0.18	21.1	1.07	2.10	0.24
**Buckwheat**	0.47	0.081	0.30	69.3	13.4	5.16	175	7.50	27.1	168	273	1.35	1.89	134	0.84	2.63	0.32	23.8	0.69	0.17	20.7	1.07	2.12	0.24
**Hemp**	0.20	0.050	0.14	27.6	9.30	2.49	95.4	2.36	11.6	69.3	152	0.61	0.62	70.9	0.37	5.97	0.12	24.4	0.32	0.073	11.1	0.45	0.90	0.10
**Hemp**	0.19	0.042	0.13	25.6	10.3	2.45	73.4	2.46	12.7	81.0	159	0.57	0.62	73.4	0.19	1.03	0.15	32.5	0.38	0.070	9.52	0.35	0.68	0.080
**Hemp**	0.18	0.045	0.13	26.5	10.3	2.45	73.6	2.48	12.7	80.7	158	0.57	0.60	73.1	0.19	1.00	0.13	32.2	0.37	0.070	9.64	0.35	0.68	0.080
**Hemp**	0.29	0.058	0.18	39.1	9.45	2.73	91.4	2.57	13.1	82.1	172	0.65	0.99	86.2	0.38	1.46	0.17	24.7	0.41	0.097	15.0	0.58	1.12	0.13
**Chenopodium**	0.23	0.054	0.14	37.9	34.9	2.60	144	8.29	27.9	194	299	0.75	1.02	159	0.48	2.03	0.17	117	0.28	0.10	9.49	0.56	1.09	0.13
**Echinochloa crusgalli**	0.91	0.10	0.52	146	8.18	4.58	95.1	1.87	10.2	57.2	108	0.84	4.09	59.5	1.21	8.04	0.58	6.41	0.77	0.34	31.3	1.63	3.19	0.35
**Echinochloa crusgalli**	0.87	0.10	0.51	150	7.18	4.27	102	1.82	10.1	54.5	107	0.84	4.08	57.5	1.09	6.76	0.58	5.91	0.78	0.34	30.2	1.75	3.42	0.37
**Setaria glauca**	0.48	0.066	0.27	71.2	5.67	2.67	71.5	1.70	9.73	59.2	94.2	0.53	2.07	45.1	0.52	3.93	0.27	6.73	0.54	0.19	17.9	0.87	1.66	0.19
**Setaria glauca**	0.48	0.064	0.26	66.9	5.72	2.84	59.5	1.58	8.88	50.8	88.6	0.51	2.01	44.7	0.51	4.24	0.25	5.43	0.50	0.19	16.5	0.84	1.64	0.18

**Vaccaria segetails**	0.37	0.073	0.20	45.0	13.7	3.77	130	7.31	20.6	152	215	1.07	1.10	163	0.52	5.36	0.22	34.5	0.56	0.11	17.1	0.70	1.41	0.16
**GSP-2**	36.4	1.51	6.95	3930	51.0	21.9	328	7.22	17.3	43.2	115	26.3	236	238	26.2	585	24.5	2.15	7.34	1.22	1360	183	441	52.6
**Standard value**	36.0	1.50	7.00	4000	52.0	24.0	320	7.30	17.0	47.0	120	24.0	238	240	26.0	550	27.0	2.10	6.75	1.20	1340	180	430	56.0
**W-2a**	9.19	0.67	34.1	6370	259	95.5	1310	43.4	75.3	107	80.0	16.8	20.9	198	20.9	92.4	8.18	0.27	2.10	0.94	173	11.3	23.4	3.05
**Standard value**	9.21	0.74	36.7	6380	268	92.0	1280	44.4	72.0	106	77.7	17.9	20.2	195	21.8	93.3	7.51	0.50	1.64	0.96	173	10.6	23.2	3.02
**AGV-2**	10.5	2.17	13.0	6170	117	17.8	784	15.7	20.1	51.2	90.3	19.6	64.9	649	20.3	226	15.4	1.85	2.06	1.17	1120	37.0	68.9	8.04
**Standard value**	10.8	2.30	13.1	6300	119	19.0	778	15.1	21.0	51.5	94.0	20.4	67.8	660	19.1	232	14.1	2.00	2.00	1.17	1130	38.2	69.4	8.17

Table S2 The element results of plant remain from Chashancun cemetery.

Samples	Nd	Sm	Eu	Gd	Tb	Dy	Ho	Er	Tm	Yb	Lu	Hf	Ta	W	Tl	Pb	Th	U	PCA 01	PCA 02	PCA 03
**Large broomcorn millet**	0.33	0.066	0.013	0.057	0.0090	0.047	0.0099	0.027	0.0041	0.025	0.0040	0.032	0.0012	0.091	0.015	2.19	0.20	0.11	-1.14	-0.61	-0.32
**Large broomcorn millet**	0.26	0.052	0.011	0.046	0.0077	0.041	0.0088	0.024	0.0037	0.023	0.0040	0.052	0.013	0.096	0.016	1.55	0.18	0.098	-1.16	-0.66	-0.28
**Large broomcorn millet**	0.29	0.060	0.012	0.064	0.012	0.080	0.018	0.053	0.0081	0.049	0.0082	0.032	0.0035	0.067	0.015	1.00	0.17	0.099	-1.09	-0.70	-0.38
**Small broomcorn millet**	0.26	0.050	0.011	0.047	0.0078	0.044	0.0090	0.025	0.0037	0.023	0.0040	0.042	0.0020	0.075	0.015	1.27	0.17	0.086	-1.24	-0.66	-0.31
**Small broomcorn millet**	0.23	0.046	0.010	0.043	0.0072	0.042	0.0085	0.023	0.0032	0.022	0.0036	0.037	0.0014	0.069	0.014	1.26	0.16	0.086	-1.27	-0.64	-0.33
**Small broomcorn millet**	0.23	0.048	0.010	0.046	0.0078	0.044	0.0093	0.025	0.0040	0.024	0.0039	0.037	0.00	0.068	0.014	1.28	0.16	0.084	-1.25	-0.64	-0.34
**Large foxtail millet**	1.13	0.23	0.047	0.20	0.031	0.18	0.037	0.10	0.015	0.095	0.015	0.15	0.023	0.20	0.034	4.14	0.59	0.26	0.39	-0.61	-0.25
**Large foxtail millet**	1.18	0.23	0.045	0.20	0.031	0.17	0.035	0.096	0.014	0.087	0.014	0.11	0.020	0.19	0.030	4.31	0.56	0.25	0.25	-0.56	-0.31
**Large foxtail millet**	1.13	0.22	0.041	0.084	0.029	0.18	0.037	0.10	0.016	0.10	0.015	0.16	0.023	0.31	0.024	3.31	0.50	0.25	0.24	-0.71	3.14
**Large foxtail millet**	1.12	0.22	0.042	0.086	0.030	0.18	0.037	0.10	0.015	0.100	0.015	0.17	0.023	0.25	0.024	3.28	0.50	0.25	0.23	-0.76	3.08
**Small foxtail millet**	1.27	0.25	0.048	0.22	0.037	0.21	0.038	0.11	0.016	0.095	0.016	0.13	0.025	0.21	0.033	4.24	0.65	0.27	0.49	-0.63	0.27
**Small foxtail millet**	1.36	0.26	0.051	0.22	0.034	0.19	0.041	0.11	0.016	0.10	0.015	0.12	0.024	0.21	0.034	4.29	0.70	0.27	0.51	-0.64	0.32
**Small foxtail millet**	1.17	0.24	0.045	0.21	0.032	0.19	0.039	0.11	0.015	0.095	0.015	0.12	0.023	0.19	0.032	6.09	0.55	0.26	0.38	-0.52	-0.17
**Small foxtail millet**	1.18	0.23	0.046	0.21	0.033	0.19	0.040	0.12	0.018	0.11	0.018	0.12	0.024	0.20	0.034	5.06	0.55	0.26	0.46	-0.47	-0.32
**Barley**	2.25	0.46	0.090	0.42	0.065	0.38	0.078	0.22	0.032	0.20	0.031	0.26	0.044	0.32	0.054	8.06	0.93	0.47	2.29	-0.16	-0.61
**Barley**	2.24	0.46	0.087	0.41	0.062	0.36	0.072	0.20	0.029	0.18	0.028	0.40	0.046	0.39	0.049	8.17	0.86	0.52	2.20	-0.11	-0.25
**Barley**	2.15	0.45	0.087	0.39	0.060	0.36	0.071	0.20	0.029	0.18	0.028	0.30	0.045	0.31	0.048	7.59	0.88	0.51	2.12	-0.13	-0.47
**Buckwheat**	1.02	0.22	0.043	0.20	0.031	0.19	0.038	0.11	0.015	0.092	0.015	0.12	0.013	0.41	0.030	5.51	0.41	0.41	0.46	1.44	-0.88
**Buckwheat**	0.90	0.19	0.039	0.18	0.029	0.17	0.034	0.099	0.014	0.084	0.014	0.092	0.012	0.39	0.029	4.86	0.40	0.40	0.28	1.40	-0.82
**Buckwheat**	0.92	0.19	0.038	0.19	0.029	0.17	0.035	0.095	0.014	0.082	0.013	0.066	0.0099	0.38	0.028	4.57	0.40	0.44	0.27	1.61	-1.06
**Hemp**	0.39	0.086	0.017	0.088	0.014	0.083	0.017	0.048	0.0070	0.045	0.0078	0.14	0.00	0.26	0.016	2.85	0.21	0.22	-0.74	0.39	-0.21

**Hemp**	0.29	0.062	0.012	0.059	0.0093	0.052	0.011	0.030	0.0042	0.027	0.0049	0.029	0.0063	0.28	0.016	1.87	0.17	0.19	-0.96	0.55	-0.10
**Hemp**	0.29	0.060	0.013	0.060	0.0093	0.054	0.011	0.031	0.0044	0.027	0.0051	0.027	0.0024	0.26	0.016	1.93	0.17	0.19	-0.97	0.55	-0.16
**Hemp**	0.46	0.098	0.019	0.094	0.014	0.084	0.018	0.050	0.0074	0.043	0.0072	0.040	0.0050	0.31	0.020	2.57	0.22	0.22	-0.67	0.52	-0.27
**Chenopodium**	0.49	0.11	0.020	0.11	0.016	0.10	0.022	0.061	0.0086	0.054	0.0090	0.054	0.0062	1.13	0.027	8.13	0.22	0.45	-0.26	3.43	2.22
**Echinochloa crusgalli**	1.27	0.25	0.051	0.24	0.038	0.22	0.045	0.13	0.019	0.12	0.018	0.20	0.030	0.21	0.038	4.11	0.62	0.28	0.68	-0.69	-0.13
**Echinochloa crusgalli**	1.34	0.26	0.052	0.22	0.036	0.21	0.042	0.12	0.017	0.11	0.018	0.17	0.035	0.20	0.036	3.99	0.64	0.28	0.64	-0.73	-0.14
**Setaria glauca**	0.67	0.13	0.026	0.11	0.018	0.11	0.022	0.064	0.0091	0.059	0.0092	0.10	0.013	0.16	0.025	2.18	0.36	0.23	-0.43	-0.38	-0.26
**Setaria glauca**	0.66	0.13	0.026	0.12	0.018	0.11	0.023	0.061	0.0090	0.057	0.0086	0.11	0.010	0.14	0.021	2.33	0.34	0.17	-0.49	-0.47	-0.26
**Vaccaria segetails**	0.61	0.13	0.027	0.13	0.019	0.11	0.023	0.064	0.0089	0.057	0.0098	0.12	0.0042	0.44	0.022	4.50	0.26	0.31	-0.21	1.58	-0.39
**GSP-2**	200	26.7	2.26	12.3	1.32	5.93	0.98	2.34	0.29	1.69	0.24	15.0	0.83	0.41	1.28	42.6	107	2.53	-	-	-
**Standard value**	190	27.0	2.20	12.0	1.32	5.86	1.0	2.40	0.28	1.60	0.24	14.0	0.90	0.39	1.28	42.0	105	2.40	-	-	-
**W-2a**	12.8	3.34	1.12	3.72	0.62	3.85	0.79	2.23	0.32	1.98	0.30	2.46	0.46	0.29	0.10	7.90	2.28	0.50	-	-	-
**Standard value**	13.1	3.30	1.10	3.71	0.63	3.79	0.81	2.27	0.33	2.05	0.31	2.45	0.47	0.30	0.10	7.83	2.26	0.51	-	-	-
**AGV-2**	29.5	5.53	1.51	4.49	0.63	3.47	0.67	1.79	0.25	1.58	0.24	5.01	0.80	0.52	0.28	13.0	5.98	1.85	-	-	-
**Standard value**	30.5	5.51	1.55	4.52	0.65	3.55	0.68	1.83	0.26	1.65	0.25	5.14	0.79	0.55	0.28	13.1	6.17	1.89	-	-	-

Table S3 Descriptive Statistics of element results of plant remains from Chashancun cemetery.

Element	N	Minimum	Maximum	Mean	Std. Deviation
Li	30	0.09	1.25	0.54	0.36
Be	30	0.04	0.16	0.08	0.04
Sc	30	0.13	0.79	0.34	0.20
Ti	30	22.30	212.00	87.54	60.01
V	30	2.21	34.90	8.64	6.08
Cr	30	1.21	7.38	3.64	1.70
Mn	30	25.10	201.00	99.74	51.52
Co	30	0.64	8.29	2.90	2.26
Ni	30	3.92	27.90	12.80	7.36
Cu	30	23.00	194.00	75.41	46.58
Zn	30	42.00	299.00	137.97	77.11
Ga	30	0.19	1.41	0.72	0.40
Rb	30	0.60	6.07	2.47	1.73
Sr	30	18.40	163.00	72.67	41.25
Y	30	0.14	2.20	0.78	0.55
Zr	30	1.00	16.70	4.77	3.67
Nb	30	0.09	0.86	0.34	0.22
Mo	30	2.36	117.00	15.19	21.84
Sn	30	0.21	1.13	0.60	0.28
Cs	30	0.07	2.69	0.41	0.65
Ba	30	6.53	52.70	21.52	13.15
La	30	0.31	2.91	1.13	0.75
Ce	30	0.58	5.56	2.22	1.48
Pr	30	0.07	0.62	0.25	0.16
Nd	30	0.23	2.25	0.90	0.59
Sm	30	0.05	0.46	0.18	0.12
Eu	30	0.01	0.09	0.04	0.02
Gd	30	0.04	0.42	0.16	0.11
Tb	30	0.01	0.07	0.03	0.02
Dy	30	0.04	0.38	0.15	0.09
Ho	30	0.01	0.08	0.03	0.02
Er	30	0.02	0.22	0.09	0.05
Tm	30	0.00	0.03	0.01	0.01
Yb	30	0.02	0.20	0.08	0.05
Lu	30	0.00	0.03	0.01	0.01
Hf	30	0.03	0.40	0.12	0.09
Ta	30	0.00	0.05	0.02	0.01
W	30	0.07	1.13	0.26	0.20
Tl	30	0.01	0.05	0.03	0.01
Pb	30	1.00	8.17	3.88	2.14
Th	30	0.16	0.93	0.42	0.24
U	30	0.08	0.52	0.26	0.13
Vilid N	30				

Table S4 Total Variance Explained of element results of plant remains from Chashancun cemetery.

Component	Initial Eigenvalues			Extration Sums of Squared Loadings		
	Total	% of Variance	Cumulative %	Total	% of Variance	Cumulative %
1	31.343	74.627	74.627	31.343	74.627	74.627
2	7.756	18.466	93.092	7.756	18.466	93.092
3	1.235	2.941	96.033	1.235	2.941	96.033
4	0.728	1.732	97.765			
5	0.393	0.936	98.701			
6	0.135	0.322	99.024			
7	0.106	0.252	99.276			
8	0.061	0.145	99.42			
9	0.055	0.13	99.551			
10	0.051	0.121	99.671			
11	0.04	0.094	99.766			
12	0.028	0.067	99.833			
13	0.018	0.044	99.876			
14	0.011	0.027	99.903			
15	0.008	0.018	99.921			
16	0.007	0.016	99.937			
17	0.006	0.014	99.951			
18	0.004	0.011	99.962			
19	0.004	0.009	99.971			
20	0.004	0.008	99.98			
21	0.003	0.006	99.985			
22	0.002	0.004	99.99			
23	0.002	0.004	99.994			
24	0.001	0.003	99.996			
25	0.001	0.002	99.998			
26	0	0.001	99.999			
27	0	0	100			
28	4.97E-05	0	100			

29	3.98E-05	9.47E-05	100
30	1.19E-15	2.84E-15	100
31	8.44E-16	2.01E-15	100
32	6.54E-16	1.56E-15	100
33	5.09E-16	1.21E-15	100
34	2.05E-16	4.88E-16	100
35	8.58E-17	2.04E-16	100
36	-1.22E-16	-2.90E-16	100
37	-3.29E-16	-7.83E-16	100
38	-4.52E-16	-1.08E-15	100
39	-5.69E-16	-1.35E-15	100
40	-8.05E-16	-1.92E-15	100
41	-9.87E-16	-2.35E-15	100
42	-1.45E-15	-3.45E-15	100

Table S5 component matrix and component coefficient matrix of element results of plant remains from Chashancun cemetery.

Element	Component matrix		component coefficient matrix	
	PC 01	PC 02	PC 01	PC 02
Li	0.959	-0.228	0.031	-0.029
Be	0.987	-0.115	0.031	-0.015
Sc	0.951	-0.278	0.030	-0.036
Ti	0.957	-0.259	0.031	-0.033
V	0.279	0.887	0.009	0.114
Cr	0.947	0.129	0.030	0.017
Mn	0.840	0.503	0.027	0.065
Co	0.403	0.880	0.013	0.113
Ni	0.530	0.823	0.017	0.106
Cu	0.391	0.906	0.012	0.117
Zn	0.506	0.838	0.016	0.108
Ga	0.774	0.452	0.025	0.058
Rb	0.944	-0.293	0.030	-0.038
Sr	0.488	0.839	0.016	0.108
Y	0.985	-0.111	0.031	-0.014
Zr	0.897	-0.144	0.029	-0.019
Nb	0.977	-0.166	0.031	-0.021
Mo	-0.078	0.896	-0.002	0.116
Sn	0.952	-0.121	0.030	-0.016
Cs	0.284	-0.285	0.009	-0.037
Ba	0.982	-0.158	0.031	-0.020
La	0.977	-0.191	0.031	-0.025
Ce	0.979	-0.185	0.031	-0.024
Pr	0.982	-0.173	0.031	-0.022
Nd	0.986	-0.152	0.031	-0.020
Sm	0.991	-0.112	0.032	-0.014
Eu	0.992	-0.116	0.032	-0.015
Gd	0.964	-0.017	0.031	-0.002
Tb	0.993	-0.093	0.032	-0.012
Dy	0.993	-0.079	0.032	-0.010
Ho	0.993	-0.072	0.032	-0.009
Er	0.991	-0.069	0.032	-0.009
Tm	0.988	-0.092	0.032	-0.012
Yb	0.987	-0.106	0.031	-0.014
Lu	0.989	-0.081	0.032	-0.010
Hf	0.897	-0.164	0.029	-0.021
Ta	0.928	-0.286	0.030	-0.037
W	0.271	0.863	0.009	0.111
Tl	0.978	-0.024	0.031	-0.003
Pb	0.860	0.396	0.027	0.051
Th	0.956	-0.246	0.030	-0.032
U	0.848	0.513	0.027	0.066

Table S6 Agglomeration Schedule of element results of plant remains from Chashancun cemetery.

Stage	Cluster Combined		Coefficients	Stage Cluster First Appears		Next Stage
	Cluster 1	Cluster 2		Cluster 1	Cluster 2	
1	5	6	0.206	0	0	2
2	4	5	0.561	0	1	13
3	22	23	2.141	0	0	20
4	11	12	2.401	0	0	12
5	9	10	6.344	0	0	10
6	2	3	12.276	0	0	9
7	26	27	80.213	0	0	21
8	16	17	83.336	0	0	15
9	1	2	89.448	0	6	13
10	7	9	111.154	0	5	18
11	8	13	209.764	0	0	16
12	11	14	238.116	4	0	16
13	1	4	258.919	9	2	27
14	28	29	269.119	0	0	23
15	15	16	278.38	0	8	28
16	8	11	424.769	11	12	18
17	18	19	542.481	0	0	19
18	7	8	587.073	10	16	21
19	18	20	748.313	17	0	24
20	21	22	757.039	0	3	22
21	7	26	895.142	18	7	23
22	21	24	950.019	20	0	25
23	7	28	5443.625	21	14	25
24	18	30	6743.362	19	0	26
25	7	21	13831.287	23	22	27
26	18	25	15109.348	24	0	28
27	1	7	20570.741	13	25	29
28	15	18	34747.649	15	26	29
29	1	15	64039.568	27	28	0

Note: 1-30 represent the 30 samples in Table S2 from top to bottom.
